# Chorea and Tick-Borne Encephalitis, Poland

**DOI:** 10.3201/eid1909.130804

**Published:** 2013-09

**Authors:** Joanna Zajkowska, Anna Moniuszko, Piotr Czupryna, Wiesław Drozdowski, Waldemar Krupa, Katarzyna Guziejko, Maciej Kondrusik, Sambor Grygorczuk, Slawomir Pancewicz

**Affiliations:** Author affiliation: Medical University, Białystok, Poland

**Keywords:** tick-borne encephalitis, TBE, encephalitis, vector-borne infections, chorea, thalamus, viruses, MRI, magnetic resonance imaging, computed tomography, CSF, cerebrospinal fluid, Poland

**To the Editor:** Tick-borne encephalitis (TBE) is caused by infection with vector-borne viruses classified in the family *Flaviviridae*. The disease is characterized by a biphasic course. In the first phase, fever, fatigue, malaise, headache, and arthralgia occur; in the second phase, neurologic signs develop, and the clinical spectrum ranges from mild meningitis to severe encephalitis, myelitis, and polyradiculitis, sometimes with a fatal outcome (*1*). The clinical signs and symptoms of TBE can be explained by affinity of TBEV to distinct regions of the CNS, such as multinodular to patchy encephalomyelitis accentuated in the spinal cord, brain stem, and cerebellum (*2*)*.* According to the European Centre for Disease Prevention and Control, a notable rise in the incidence of TBE has been observed in Europe and Asia in recent years, and the disease is considered a growing public health concern in many parts of the world (*3*). In Poland, an average of 250 TBE cases are reported each year. Mean incidence is 0.75 cases/100,000 population, and incidence is highest in the northeastern part of the country, which is considered to be a TBE-endemic area (11.53 cases/100,000 inhabitants).

Chorea is defined as a state of excessive, spontaneous movements that are irregularly timed, nonrepetitive, randomly distributed, and abrupt in character (http://emedicine.medscape.com/article/1149854)*.* This condition is a result of absent subthalamic nucleus inhibition, which increases motor activity through the motor thalamus. It is a dominant symptom of Huntington’s disease, which is an inherited, progressive, neurodegenerative disorder. Nonhereditary causes of chorea, such as infectious chorea in the course of acute manifestations of bacterial or aseptic meningitis or encephalitis, have been described (*4*,*5*)*.* We describe a case of chorea in the course of TBE in a man in Poland.

A 38-year-old man was admitted to the hospital in Białystok, Poland, because of fever (40°C), headache, vomiting, and drowsiness that had lasted for 1 week. The patient was a forest worker who reported frequent tick bites; for religious reasons, he had not been vaccinated against TBE. Physical examination revealed palsy in the seventh facial nerve, tremor in the facial muscles on the left side, muscle weakness in the left upper and lower limbs, and positive meningeal signs. Testing of cerebrospinal fluid (CSF) revealed inflammatory features (cytosis, 63 cells/µL, and protein concentration 68 mg/dL). Immunologic test results showed CSF IgM of 9.04 IU/mL and IgG of 55.1 IU/mL against TBE virus (cutoff 1.4 for IgM, 7.0 for IgG). Computed tomography scan and magnetic resonance imaging (MRI) of the brain revealed widening of ventricles. The patient was treated with mannitol, dexamethasone, third-generation cephalosporin, and diazepam; after signs and symptoms improved, the patient was discharged home on his request.

Eleven days after discharge, the patient was readmitted to the hospital because of deteriorating clinical signs (progressive muscle weakness and excessive tremor of the left-side limbs). Intensive propulsion, involuntary muscle movements (limbs and face), muscle weakness of left upper and lower limbs, and “walk dance” were observed (Video). Results of CSF analysis showed continued inflammatory features (pleocytosis, 64 cells/µL, and protein concentration 119.4 mg/dL). Results of testing for antibodies against TBE virus in CSF and serum were strongly positive (CSF IgM >14 U/mL, IgG 107 U/mL; serum IgM 7.92 U/mL, IgG 87.6 U/mL). Test results for *Borrelia burgdorferi* were negative 3 times. MRI of the brain showed lesions in the lenticular nucleus, caudate nucleus on the right side, and internal capsules of both sides (Figure). 

**Video:**
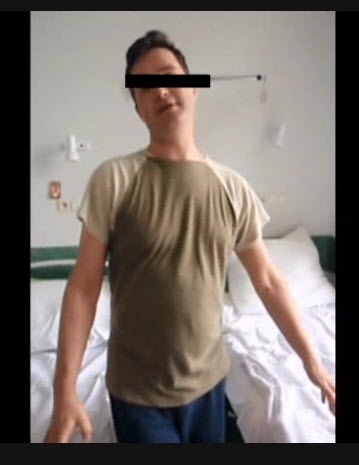
Intensive propulsion, involuntary muscle movements (limbs and face), muscle weakness of left upper and lower limbs, and “walk dance” in 38-year-old man with tick-borne encephalitis and chorea, Poland.

**Figure:**
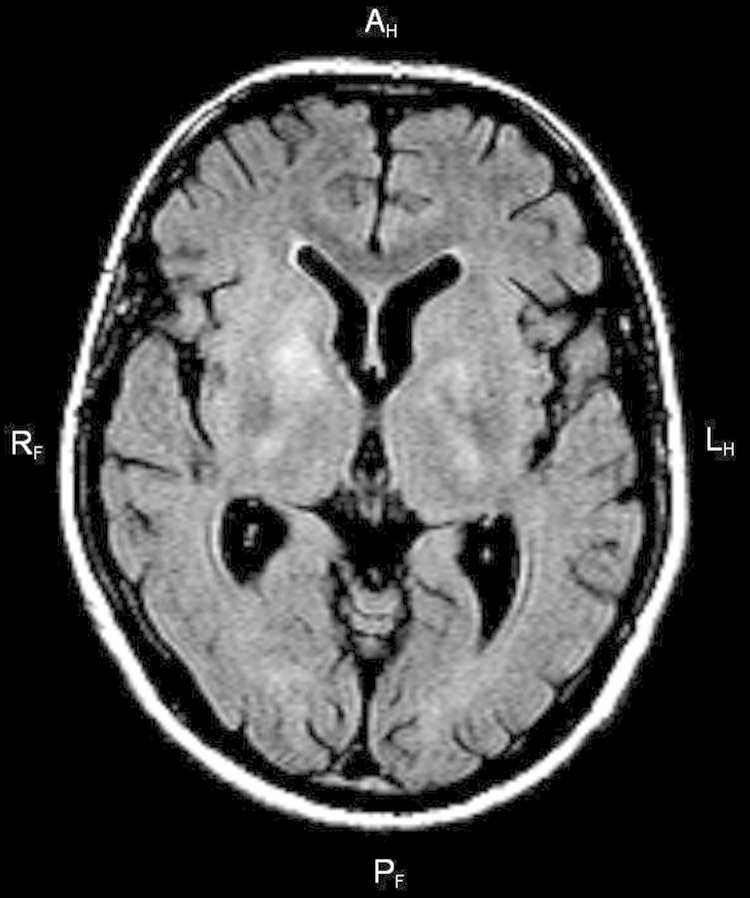
Fluid-attenuated inversion recovery magnetic resonance imaging of the brain of a 38-year-old man with tick-borne encephalitis and chorea. The image shows bilateral areas of hyperintensity in T2, affecting the nucleus caudate, internal capsule, and thalami.

After neurologic and psychiatric consultations, chorea in the course of TBE was diagnosed. The patient was treated with haloperidol, dexamethasone, pentoxifylline, nitrazepam, and midazolam, and his signs and symptoms improved.

One month later, follow-up testing and examination were performed. No abnormalities on physical examination or laboratory test results were observed except pleocytosis in CSF (35 cells/µL). The patient reported aphasia and deterioration of memory. Six months later, results of CSF testing were within normal limits, and repeat MRI showed resolution of inflammatory lesions.

In this case, chorea occurred in the course of TBE. The neurologic phase of TBE was not preceded by the expected first-phase signs and symptoms. The diagnosis was confirmed with serologic test results, which were strongly positive in serum and CSF, and virus neutralization testing performed as described in Stiasny et al. (*6*) showed a strongly positive titer of 1:320. We excluded hepatitis C virus; West Nile virus infection has not been reported in Poland. Because of his religious beliefs, the patient had not been vaccinated against TBE or yellow fever; he had not traveled abroad and had not been exposed to dengue virus. The differential diagnosis included Lyme disease, but the results of repeated serologic tests for *B. burgdorferi* were negative.

Chorea arises deep in the basal ganglia; high-definition MRI demonstrates caudate atrophy (*7*)*.* In patients with TBE, MRI may reveal unilateral or bilateral thalamus lesions, as well as lesions in the cerebellum, brainstem, and nucleus caudalis (*8*)*.* One case study presented a case of simultaneous lesions in the thalamus, stem, and spinal cord (*9*)*.* Chorea developed in the patient reported here because of inflammatory lesions in the region of the thalamus. Supportive care is standard for patients with chorea (*10*), but after treatment for TBE, the lesions in this patient resolved, and the chorea abated.
